# EDTA and Taurolidine Affect Pseudomonas aeruginosa Virulence *In Vitro*—Impairment of Secretory Profile and Biofilm Production onto Peritoneal Dialysis Catheters

**DOI:** 10.1128/Spectrum.01047-21

**Published:** 2021-11-17

**Authors:** Bruna Colombari, Gaetano Alfano, Christian Gamberini, Gianni Cappelli, Elisabetta Blasi

**Affiliations:** a Microbiology and Virology Laboratories, University of Modena and Reggio Emilia, Modena, Italy; b Nephrology, Dialysis and Renal Transplant Unit, University Hospital of Modena, Modena, Italy; c School of Specialization in Microbiology and Virology, University of Modena and Reggio Emilia, Modena, Italy; d Department of Surgery, Medicine, Dentistry and Morphological Sciences with interest in Transplant, Oncological and Regenerative Medicine, University of Modena and Reggio Emilia, Modena, Italy; University of Guelph

**Keywords:** lock solutions, *Pseudomonas aeruginosa*, biofilm, peritoneal dialysis catheters

## Abstract

Peritoneal catheter-associated biofilm infection is reported to be the main cause of refractory peritonitis in peritoneal dialysis patients. The application of antimicrobial lock therapy, based on results on central venous catheters, may be a promising option for treatment of biofilm-harboring peritoneal catheters. This study investigated the effects of two lock solutions, EDTA and taurolidine, on an *in vitro* model of Pseudomonas aeruginosa biofilm-related peritoneal catheter infection. Silicone peritoneal catheters were incubated for 24 h with a bioluminescent strain of P. aeruginosa. Then, serial dilutions of taurolidine and/or EDTA were applied (for 24 h) once or twice onto the contaminated catheters, and P. aeruginosa viability/persistence were evaluated in real time up to 120 h using a Fluoroskan reader. On selected supernatants, high-performance liquid chromatography mass spectrometry (HPLC-MS) analysis was performed to measure the production of autoinducers (AI), phenazines, and pyocyianines. Taurolidine alone or in combination with EDTA caused a significant decrease of bacterial load and biofilm persistence on the contaminated catheters. The treatment did not lead to the sterilization of the devices, yet it resulted in a substantial destructuration of the catheter-associated P. aeruginosa biofilm. HPLC-MS analysis showed that the treatment of biofilm-harboring catheters with taurolidine and EDTA also affected the secretory activity of the pathogen. EDTA and taurolidine affect P. aeruginosa biofilm produced on peritoneal catheters and profoundly compromise the microbial secretory profile. Future studies are needed to establish whether such lock solutions can be used to render peritoneal catheter-related infections more susceptible to antibiotic treatment.

**IMPORTANCE** An *in vitro* model allows studies on the mechanisms by which the lock solutions exert their antimicrobial effects on catheter-associated biofilm, thus providing a better understanding of the management of devise-associated infections.

## INTRODUCTION

Peritoneal dialysis (PD)-associated peritonitis is the most concerning complication in PD patients (1). It carries significant morbidity, mortality, increase in treatment cost, and early failure of peritoneal ultrafiltration (2–4). The most common pathogens leading to peritonitis are Gram-positive bacteria that colonize human skin and hands, but several other organisms, including Gram-negative bacteria, have been involved (4, 5). The source of infection for the vast majority of PD-related cases of peritonitis is the PD catheter (6). It provides bacteria with a gateway to the peritoneal cavity, a hospitable environment characterized by few host-defense cells (macrophage) and humoral factors (immunoglobulins and complement). Moreover, the sterile and warm glucose-based dialysis fluid used for peritoneal dialysis represents an ideal milieu for rapid bacterial growth.

Peritonitis is generally easily treated with intraabdominal antibiotics, but severe or relapsing episodes of peritonitis, such as those caused by Pseudomonas aeruginosa frequently result in catheter removal and temporary hemodialysis treatment (7). Such infections arise from bacterial adhesion to biomaterial surfaces with subsequent formation of antibiotic-refractory biofilms that are challenging to handle with *in situ* intraperitoneal antibiotics. *Ex vivo* studies of PD catheters removed from patients experiencing peritonitis showed that such catheters are covered by microbial biofilms usually composed of a single species or mixed microbial populations (8–10). The abundant extracellular polymeric matrix embedding these microbial communities leads to inadequate penetration into biofilms of standard intraperitoneal antibiotics, resulting in a high rate of relapsing infections which are treated only by catheter removal. The use of high concentrations of an antibiotic in the catheter lumen (lock solution), while not clinically used in routine yet, is a highly attractive option for prevention or treatment of peritoneal catheter-related infections. Most studies in the field of catheter-related infections have focused on the use of antibiotic lock treatment as a therapeutic option for intravascular catheter-related infections. Currently, only a few studies have investigated the effects of lock therapy on microbial biofilms in PD (8–10). In hemodialysis, antimicrobial lock solution has been proven to be such an effective adjunctive treatment for biofilm-associated central venous catheter-related infections (11–14) that lock solution is nowadays the recommended rescue procedure for colonized intravascular catheters (15).

Taurolidine and calcium disodium EDTA are the most frequently prescribed antimicrobial lock solutions due to broad-spectrum activity and lack of bacterial resistance compared to standard antibiotic solutions. Promising anecdotal evidence provides clues on the successful treatment of relapsing peritonitis with taurolidine catheter-locking solution in peritoneal dialysis patients (16–18). Taurolidine has been effective in achieving the eradication of P. aeruginosa in PD patients with relapsing peritonitis (17, 18). There is limited experience with the use of the metal chelator EDTA as a lock solution in PD dialysis, but *in vitro* studies report an effective antibacterial effect of EDTA when used alone or in combination with classic antibiotics in models of P. aeruginosa infections (19–21) and P. aeruginosa biofilm-associated infections (22–24).

The Gram-negative bacterium P. aeruginosa is an opportunistic highly redoubtable pathogen, especially in fragile patients such as PD patients; often, P. aeruginosa is responsible for biofilm-associated infections when medical devices such as catheters, mechanical ventilation, and heart valves have to be used (25). In P. aeruginosa infections not only biofilm formation, but a series of other virulence factors such as proteases, toxins, extracellular polysaccharides, yellow-green siderophores (pyoverdine), and phenazines/pyocyanins play a crucial role. Many of these virulent tracts are under the control of quorum sensing (QS) (26), a communication mechanism that regulates gene expression in response to cell density. In P. aeruginosa, four QS systems have been identified, each acting through its own signal molecules, called autoinducers (AI) (27). These AI, indicated by the abbreviations 3-oxo-C12-HSL (3-oxododecanoyl-l-homoserine lactone), C4-HSL (*N*-butanoyl homoserine lactone), PQS (2-heptyl-3hydroxy-4-quinolone), and IQS (integrated quorum sensing: 2-2-hydroxy-phenyl-thiazole-4-carbaldehyde), greatly affect Pseudomonas virulence; indeed, they contribute to microbial adhesion, colonization, biofilm formation, microbial dissemination/dispersion, tolerance to immune cells, and resistance to drugs and detergents, determining the course and outcome of the disease (27–29). Moreover, AI influence the release of siderophores, such as pyoverdine, involved in iron removal from the environment, and phenazines, whose role in microbial adhesion and biofilm thickness and biomass increase have been reported (30, 31). The AI are also involved in oxidative stress; in fact, they interact with molecular oxygen to form reactive oxygen species, such as H_2_O_2_, which modify the redox balance, causing cell injury and death (32). Recently, we focused on Pseudomonas as a prototype difficult-to-counteract pathogen, due to its various virulence factors, including the ability to tightly form biofilm onto abiotic surfaces, such as endotracheal tubes (33). Using an engineered strain, whose viable cells emit a bioluminescence signal (BLI) readily measurable in real time, *in vitro* models can be promptly set up as miniaturized, easy-to-use, and highly sensitive systems (34, 35).

Here, we allowed BLI-P. aeruginosa biofilm production on peritoneal dialysis catheters (PDC) and assessed the possible counteracting effects of EDTA and taurolidine used as lock solutions; biofilm formation and persistence have been investigated, as well as P. aeruginosa secretory profile. The *in vitro* efficacy of EDTA and taurolidine opens up their possible clinical use to mitigate P. aeruginosa virulence in critical settings such as PD patients.

## RESULTS

### EDTA and taurolidine effects on planktonic BLI-Pseudomonas cells.

Initially, to establish the antimicrobial activity of taurolidine against BLI-Pseudomonas planktonic cells, we employed the standardized CLSI method (36). Accordingly, 10 different dilutions of taurolidine were assessed for antimicrobial activity using the microdilution method. As assessed by visual observation after 24 h of incubation at 37°C, the MIC was established at 0.0312%.

Subsequently, we employed a previously established BLI-based assay (37) to assess in real time the effects of the two lock solutions on P. aeruginosa planktonic cells; serial drug dilutions were prepared to obtain the final concentrations of 2.5%, 0.75%, and 0.25% for EDTA and 0.5%, 0.25%, and 0.125% for taurolidine. In particular, the highest dose of taurolidine employed in the present study was approximately 20-fold the MIC value (data not shown). As depicted in Table 1, after 1 h of treatment, a significant decrease of the relative luminescence units (RLU) levels was observed in taurolidine-treated samples, to a similar extent for all tested doses; in contrast, EDTA had little or no effect, causing a partial RLU reduction with respect to the untreated samples (SB) only at a 2.5% concentration. After 6 h and 24 h of treatment (Table 1), the RLU drastically decreased at all conditions tested, with EDTA at the lowest dose (0.25%) being the less effective one.

**TABLE 1:** EDTA and/or Taurolidine effects on planktonic BLI-Pseudomonas cells^*a*^

Treatment	RLU at:^*b*^		
	1 h	6 h	24 h
SB	0.0024 ± 0.0003	0.9310 ± 0.0133	18.8325 ± 0.3815
EDTA 2.5%	0.0017 ± 0.0001	0.0034 ± 0.0006***	0.0001 ± 0.0001***
Tauro 0.5%	0.0008 ± 0.0002 *	0.0004 ± 0.0001***	0.0001 ± 0.0002***
EDTA 2.5% + Tauro 0.5%	0.0005 ± 0.0001*	0.0006 ± 0.00003***	0.0007 ± 0.0005***
EDTA 0.75%	0.0023 ± 0.0002	0.0070 ± 0.0005***	0.0867 ± 0.0805***
Tauro 0.25%	0.0005 ± 0.0001*	0.0007 ± 0.0001***	0.0029 ± 0.0007***
EDTA 0.75% + Tauro 0.25%	0.0004 ± 0.0001*	0.0013 ± 0.0003***	0.0197 ± 0.0018***
EDTA 0.25%	0.0022 ± 0.0001	0.1296 ± 0.0079***	5.6897 ± 0.3240***
Tauro 0.125%	0.0002 ± 0.0001*	0.0027 ± 0.0002***	0.0357 ± 0.0039***
EDTA 0.25% + Tauro 0.125%	0.0005 ± 0.0001*	0.0065 ± 0.0003***	0.0598 ± 0.0051***

a BLI-Pseudomonas (10^5^/ml; 100 μl/well) was exposed to the indicated doses of EDTA and/or taurolidine or SB (untreated control); after 1 h, 6 h, and 24 h, the BLI signal was measured. The values were expressed as the mean ± SEM of the RLU from six replicates obtained in two independent experiments. Statistical analysis was performed according to Student’s *t* test using GraphPad Prism 8.

b *, *P* < 0.05; *** *P* < 0.001.

### EDTA and taurolidine effects on a 24-h-old biofilm.

**FIG 1:**
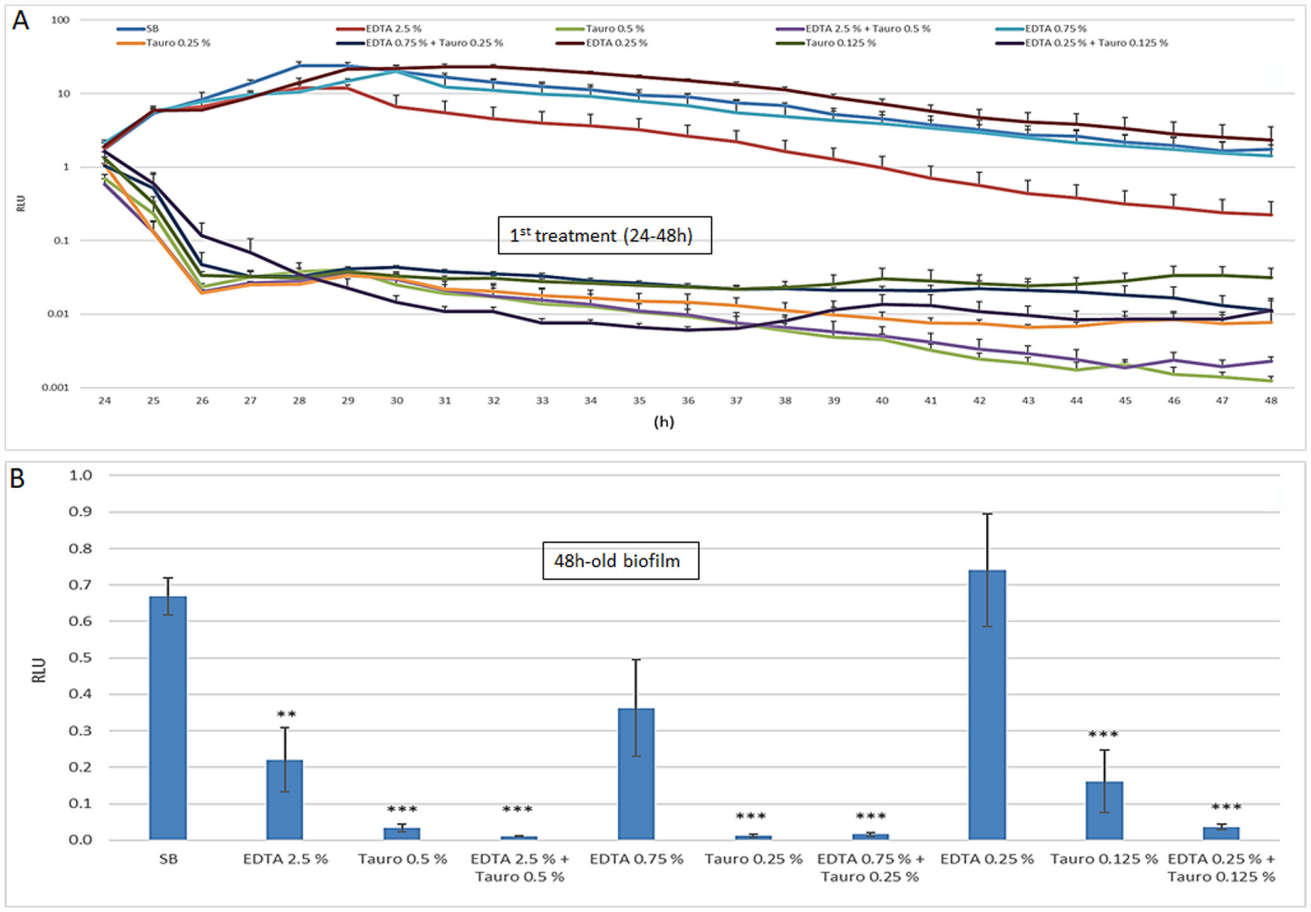
EDTA and/or taurolidine effects on a PDC-associated biofilm: microbial growth during treatment and biofilm production at time 48 h. PDC pieces (0.5 cm) were contaminated with BLI-Pseudomonas (10^5^/ml) for 24 h. Then, the PDC-associated biofilm (24 h-old) was exposed to EDTA and/or taurolidine at the indicated doses from 24 h to 48 h, at 37°C. During such incubation time, the BLI signal was recorded (A) and, at the end of the treatment, the PDC were washed again and the persistent 48-h-old biofilm was assessed (B). The values were expressed as the mean ± SEM of the RLU of 8 replicates obtained in two independent experiments. Statistical analysis was performed according to Student’s *t* test using GraphPad Prism 8. **, *P* < 0.005; ***, *P* < 0.001.

According to a previously established protocol (33), we initially assessed the ability of Pseudomonas to adhere to and produce biofilm on silicone PDC. Briefly, 0.5-cm catheter pieces were contaminated (10^4^ cells/PDC) and incubated at 37°C; then, microbial adhesion (90 min), microbial growth (0 to 24 h), and biofilm formation (24 h) were measured. As established by RLU determination, BLI-Pseudomonas efficiently adhered to the catheter pieces; also, microbial growth and PDC-associated biofilm production significantly occurred, reaching a level of RLU of 2.14 ± 0.01/PDC at time 24 h, corresponding to 9.8 × 10^8^ CFU/ml, as derived from the reference curve (data not shown). On these bases, the 24-h-old/PDC-associated biofilm was exposed to EDTA and/or taurolidine and kinetically checked for microbial growth for an additional 24 h. As shown in Fig. 1A, EDTA inhibited microbial growth only after 24h and at the highest dose (2.5%); under such conditions, an approximately 1-log decrease in RLU was observed with respect to the untreated control. In contrast, taurolidine allowed a rapid (2 h) reduction in RLU levels, which persisted throughout the 24-h observation period. In particular, compared to the control, the BLI signal dropped of about 3 log in taurolidine samples at 0.5% and slightly less (2.5 log) at the other taurolidine concentrations. Similar results were observed using taurolidine alone or in combination with EDTA. When those same samples were tested for the presence of biofilm at time 48 h, some differences among the groups were observed. As detailed in Fig. 1B, EDTA limited biofilm levels in a dose-dependent manner (2.5% and 0.75% were able to reduce biofilm by 68% and 52%, respectively; only the former produced a significant difference). Instead, taurolidine, either alone or with EDTA, massively reduced biofilm down to barely detectable levels at all the tested concentrations.

### EDTA and taurolidine effects on regrowth (48 to 72 h) and persistence (72 h) of PDC-associated biofilm.

**FIG 2:**
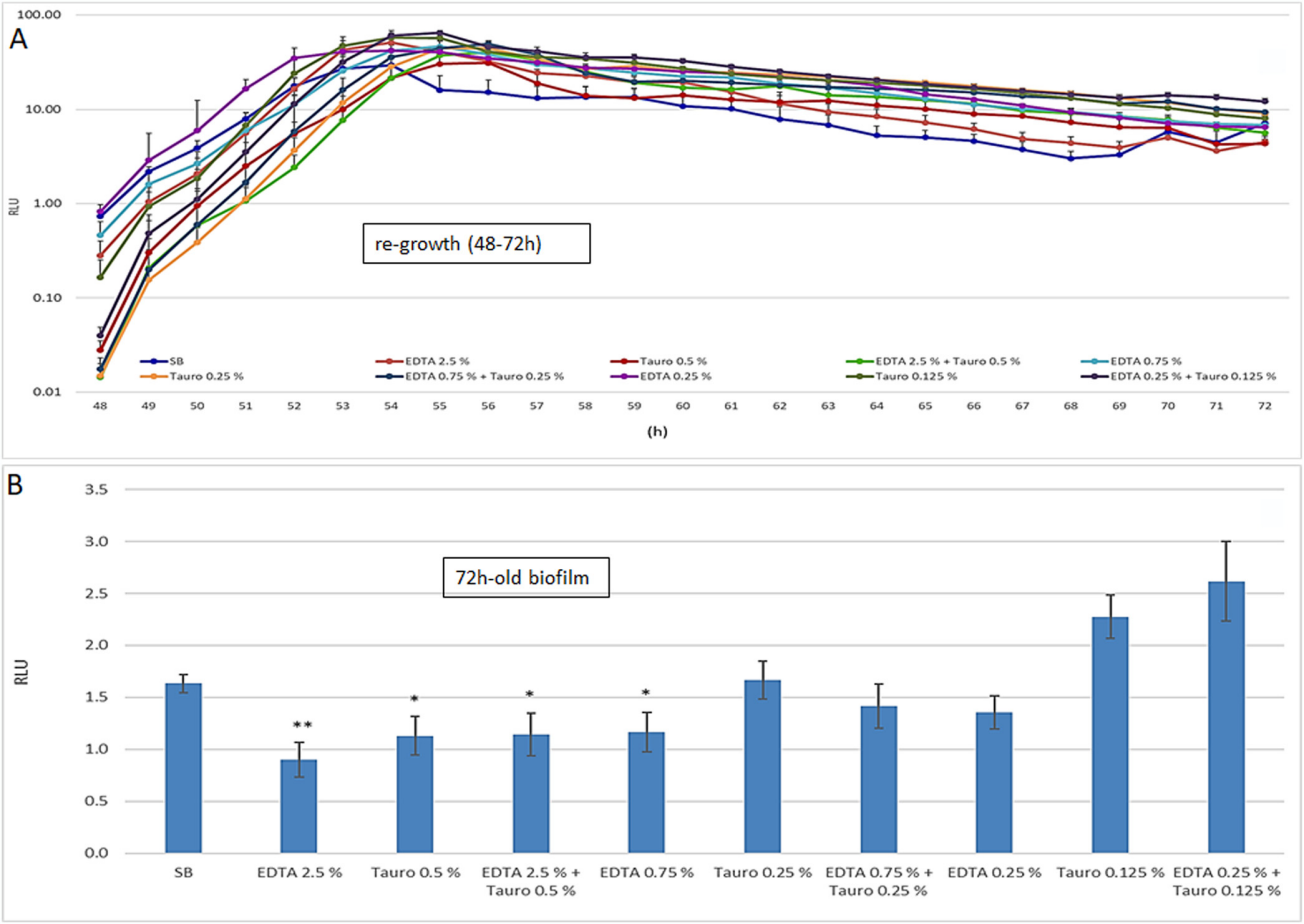
Microbial regrowth after the 1st treatment and biofilm persistence at 72 h. At time 48 h, namely, at the end of the 1st treatment, the PDC were suspended in fresh medium, incubated at 37°C from 48 h to 72 h and kinetically checked for microbial regrowth by RLU measurement (A). At time 72 h, the PDC were washed again, and the RLU was measured to quantify the persistent 72-h-old biofilm (B). The values were expressed as the mean ± SEM of the RLU of 8 replicates obtained in two independent experiments. Statistical analysis was performed according to Student’s *t* test using GraphPad Prism 8. *, *P* < 0.05; **, *P* < 0.005.

Next, we evaluated whether the EDTA and/or taurolidine inhibitory effects on Pseudomonas growth and biofilm persisted over time, focusing on the 48- to 72-h time frame. Thus, at 48 h, the lock solutions were removed, fresh medium was added to each well containing the PDC pieces and the plate was further incubated at 37°C; microbial growth was kinetically checked (48 to 72 h) by measuring the RLU. As shown in Fig. 2A, all the groups rapidly regrew in a consistent manner, reaching RLU plateau levels comparable to each other in a few hours (in about 6 h of incubation). Furthermore, at the end of that reading time, each PDS piece was washed and measured for BLI to quantify the residual biofilm (72-h-old biofilm). As shown in Fig. 2B, we observed only partially inhibitory effects at the highest doses of either EDTA or taurolidine. At the lowest dose, taurolidine treatment enhanced biofilm formation.

### Effects of EDTA and taurolidine retreatment (72 to 96 h) on PDC-associated biofilm.

**FIG 3:**
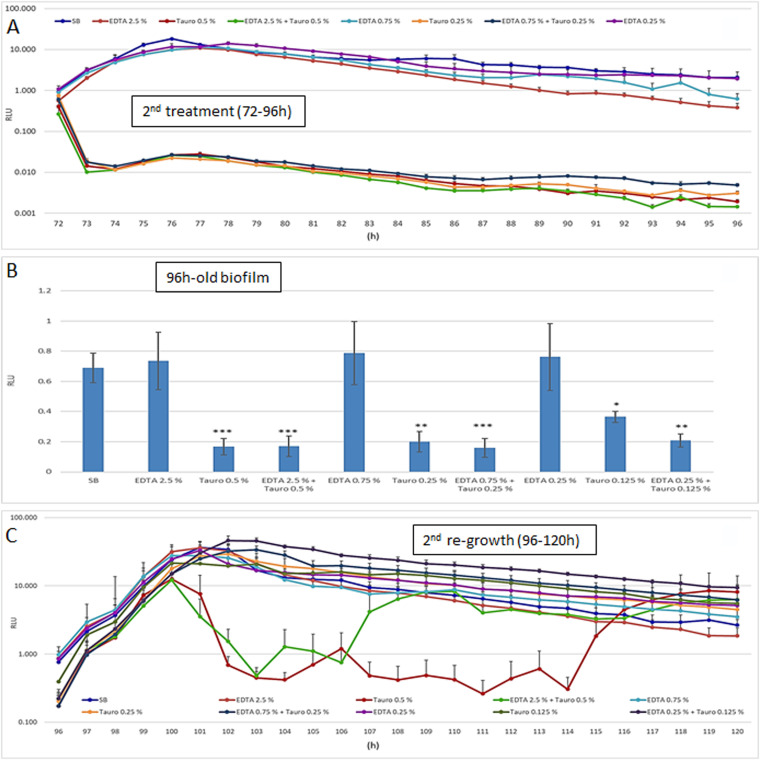
Effects of the 2nd treatment with EDTA and/or taurolidine: microbial growth (72 to 96 h) in the presence of the lock solutions, biofilm production (96 h), and regrowth after removal of EDTA and/or taurolidine (96 to 120 h). At time 72 h, the PDC pieces were subjected to a 2nd treatment with ETDA and/or taurolidine, at the indicated doses, at 37°C. During such incubation time (72 to 96 h), the BLI signal was kinetically recorded (A); at time 96 h, the PDC were washed, and the 96-h-old biofilm was assessed (B). After that, the PDC suspended in fresh medium were incubated from 96 to 120 h to assessed the regrowth (C). The values were expressed as the mean ± SEM of the RLU of 8 replicates obtained in two independent experiments. Statistical analysis was performed according to Student’s *t* test using GraphPad Prism 8. *, *P* < 0.05; **, *P* < 0.005; ***, *P* < 0.001.

With the aim of establishing whether a 2nd treatment would strengthen the inhibitory effects of EDTA and/or taurolidine on PDC-associated biofilm, the contaminated catheters were treated twice and further assessed by RLU measurement for an additional 24 h (operationally from time 72 h to 96 h). The results, shown in Fig. 3A, indicated that EDTA-treated samples returned RLU values similar to those of the control under all the working conditions and at all the time points tested. In contrast, taurolidine caused a drastic and rapid RLU reduction of about 2 log within about 1 h of treatment when used at 0.5% or 0.25% (either alone or with EDTA); such values tended to further decrease, achieving an approximately 3-log difference with respect to the controls (SB), at the latest times.

At time 96 h, each PDC piece was gently washed, immersed in fresh TSB, and assessed for residual biofilm (Fig. 3B). Also at this time point, the RLU values measured in EDTA-treated samples were similar to those of the control at all the working conditions. Once again, taurolidine treatment alone reduced the RLU in a slightly dose-dependent manner with reductions of 75%, 71%, and 47% when using 0.5%, 0.25%, and 0.125% doses, respectively; the combination of taurolidine plus EDTA showed a similar reduction.

Finally, the PDC were incubated again at 37°C and checked for further growth up to 120 h (Fig. 3C). Most of the curves were superimposable to each other, showing an initial increment of the bioluminescent signal during the first 4 h; the only exception was 0.5% taurolidine with/without EDTA, which allowed a drop of about 2 log; then, the RLU of the two groups increased again, reaching the levels of the other samples, though with a slightly different timing.

### EDTA and/or taurolidine effects on the secretory activity of PDC-associated P. aeruginosa biofilm.

Finally, we assessed the effects of EDTA and taurolidine on the ability of PDC-associated Pseudomonas biofilm to produce specific secretory products, such as several autoinducers (AI), pyocianins, and pyoverdins, known to be involved in biofilm formation/persistence and virulence (29–32). Thus, HPLC-MS analysis was performed in cell-free supernatants from PDC-associated biofilm, treated or not with EDTA and/or taurolidine. By chromatogram analysis, the different elution peaks and their areas used for semiquantitative evaluation of the specific products were identified, as previously detailed (34). Table 2 shows the peak areas and the fold changes in the production of the four AI, namely, 3-oxo-C_12_-HSL, C_4_-HSL, PQS, and IQS; the analysis was performed after the 1st treatment (upper part of the table), after the 2nd treatment (lower part), and in between (middle part of the table). A consistent presence of all such AI was observed in the supernatants of untreated biofilm (SB). After the 1st and 2nd treatments, the levels of 3-oxo-C_12_-HSL, C_4_-HSL, and PQS were affected, showing a fold change ranging from 0.77 (23% decrease) to 0.001 (99.9% decrease); moreover, the IQS levels dropped to very low or even undetectable levels.

**TABLE 2:** Secretory profile of BLI-Pseudomonas undergoing two treatments with EDTA/taurolidine (assessment of the four autoinducers [AI] by HPLC-MS)^*a*^^,^^*b*^^,^^*c*^

	3-oxo-C12-HSL		C4-HSL		PQS		IQS	
Treatment	Peak area	Fold change treated vs untreated	Peak area	Fold change treated vs untreated	Peak area	Fold change treated vs untreated	Peak area	Fold change treated vs untreated
24-h-old biofilm								
TSB	1.14E-06		2.73E-07		1.63E-09		7.10E-03	
1st treatment								
SB	1.04E-06	1	2.54E-07	1	1.91E-09	1	2.97E-06	1
EDTA 2.5%	8.02E-05	0.77	5.60E-06	0.22	2.40E-05	0.001	ND	
Tauro 0.5%	3.45E-05	0.33	3.20E-06	0.13	3.67E-07	0.19	ND	
EDTA 2.5% + Tauro 0.5%	ND		2.50E-06	0.10	1.54E-07	0.08	ND	
Regrowth								
SB	2.17E-06	1	2.90E-07	1	4.68E-09	1	5.08E-06	1
EDTA 2.5%	2.43E-06	1.1	2.85E-07	1.00	3.97E-09	0.85	4.11E-06	0.82
Tauro 0.5%	2.75E-06	1.3	2.55E-07	0.89	4.09E-09	0.87	3.49E-06	0.70
EDTA 2.5% + Tauro 0.5%	2.67E-06	1.2	2.34E-07	0.82	3.93E-09	0.84	3.66E-06	0.73
2nd treatment								
SB	1.32E-06	1	2.07E-07	1	8.90E-08	1	4.82E-06	1
EDTA 2.5%	1.04E-06	0.79	2.36E-07	1.1	1.04E-09	1.17	5.22E-06	0.3
Tauro 0.5%	1.27E-06	0.96	1.47E-06	0.1	ND		ND	
EDTA 2.5% + Tauro 0.5%	3.42E-05	0.26	1.25E-06	0.1	4.95E-07	0.06	ND	

a Supernatants from PDC pieces, contaminated and treated or not treated with EDTA and/or taurolidine, were tested by HPLC-MS. The indicated AI were investigated.

b By chromatogram analysis, the different elution peaks were identified, and their areas were used for semiquantitative evaluation of each specific product. The fold change was calculated with respect to the corresponding SB control. The results shown are from a pool of three replicates of a representative experiment out of two performed.

c ND, not detectable.

Next, the levels of four phenazines (1-hydroxidyphenazine, phenazine 1-carboxamide, phenazine-1-carboxylic acid, and pyocianin) were assessed. Table 3 shows that such molecules, abundantly found in the supernatants of the untreated controls (SB), drastically decreased to very low or undetectable levels, whether 1 or 2 treatments had been performed (upper and lower parts of the table, respectively). Furthermore, Table 4 shows the results of pyoverdine release. Also, in this case, the peak areas and the fold change of the four most abundantly detected pyoverdines (Succ-p-Ser-Y, Succa-P-Ser-Y, PyE, and PyD) dropped to undetectable levels in most cases, whether 1 or 2 treatments had been done (upper and lower parts of the table, respectively). Furthermore, as shown in the middle part of Tables 2, 3, and 4, all the above-mentioned secretory products were tested during the regrowth phase, between the 1st and the 2nd treatments (samples incubated in fresh medium from 48 to 72 h). When assessing the AI and phenazine levels (Table 2 and 3), we observed partial recovery in all the samples; in contrast, the pyoverdines consistently remained at low or undetectable levels (Table 4).

**TABLE 3:** Secretory profile of BLI-Pseudomonas undergoing two treatments with EDTA/taurolidine (assessment of four different phenazines by HPLC-MS)^*a*^^,^^*b*^^,^^*c*^

Treatment	1-Hydroxidyphenazine		Phenazine 1-carboxamide		Phenazine-1-carboxilic acid		Pyocianin	
	Peak area	Fold change treated vs untreated	Peak area	Fold change treated vs untreated	Peak area	Fold change treated vs untreated	Peak area	Fold change treated vs untreated
24-h-old biofilm (0–24 h)								
TSB	1.26E-06		1.00E-06		8.58E-08		1.24E-09	
1st treatment (48–72 h)								
SB	1.39E-06	1	2.49E-05	1	9.23E-08	1	1.84E-09	1
EDTA 2.5%	ND		ND		4.13E-07	0.045	8.07E-07	0.044
Tauro 0.5%	ND		ND		2.47E-07	0.027	1.10E-07	0.006
EDTA 2.5% + Tauro 0.5%	ND		ND		2.28E-07	0.025	4.67E-07	0.025
Regrowth (72–96 h)								
SB	2.36E-06	1	2.34E-06	1	8.70E-08	1	2.18E-09	1
EDTA 2.5%	2.36E-06	1	1.95E-06	0,83	9.50E-08	1.1	1.73E-09	0.79
Tauro 0.5%	2.25E-06	0.95	1.67E-06	0,71	8.32E-08	1	1,46E-09	0.67
EDTA 2.5% + Tauro 0.5%	2.28E-06	0.97	ND		9.12E-08	1	1.78E-09	0.82
2nd treatment (96–120 h)								
SB	2.57E-06	1	1.69E-06	1	1.54E-09	1	1.53E-09	1
EDTA 2.5%	1.08E- 06	0.42	1.18E-06	0,70	6.79E-08	0.44	6.57E-08	0.43
Tauro 0.5%	ND		3.22E-05	0,19	2.64E-07	0.02	ND	
EDTA 2.5% + Tauro 0.5%	ND		5.30E-05	0,31	2.85E-07	0.02	ND	

a Supernatants from PDC pieces, contaminated and treated or not treated with EDTA and/or taurolidine as detailed above, were tested by HPLC-MS.

b The indicated phenazines were investigated. Using chromatogram analysis, the different elution peaks were identified, and their areas used for semiquantitative evaluation of each specific product. The fold change was calculated with respect to the corresponding SB control. The results shown are from a pool of three replicates of a representative experiment out of two performed.

c ND, not detectable.

**TABLE 4:** Secretory profile of BLI-Pseudomonas undergoing two treatments with EDTA/taurolidine (assessment of four different pyoverdines by HPLC-MS)^*a*^^,^^*b*^^,^^*c*^

Treatment	Succ-p-Ser-Y		Succa-P-Ser-Y		PyE		PyD	
	Peak area	Fold change treated vs untreated	Peak area	Fold change treated vs untreated	Peak area	Fold change treated vs untreated	Peak area	Fold change treated vs untreated
24-h-old biofilm								
TSB	1.96E- 04		5.20E-04		1.00E-06		2.70E-05	
1st treatment								
SB	1.22E-04	1	5.90E-04	1	1.05E-06	1	5.08E-05	1
EDTA 2.5%	ND		ND		ND		7.40E-04	0.15
Tauro 0.5%	ND		ND		ND		ND	
EDTA 2.5% + Tauro 0.5%	ND		ND		ND		ND	
Regrowth								
SB	2.12E-04	1	7.77E-04	1	3.08E-06	1	1.46E-06	1
EDTA 2.5%	1.97E-04	0.9	ND		ND		7.90E-05	0.54
Tauro 0.5%	ND		ND		6.70E-05	0,22	8.14E-05	0.56
EDTA 2.5% + Tauro 0.5%	ND		ND		ND		6.60E-05	0.45
2nd treatment								
SB	9.30E-03	1	1.07E-04	1	1.61E-05	1	7.16E-05	1
EDTA 2.5%	ND		ND		ND	ND	ND	
Tauro 0.5%	ND		ND		ND	ND	ND	
EDTA 2.5% + Tauro 0.5%	ND		ND		ND	ND	ND	

a Supernatants from PDC pieces, contaminated and treated or not treated with EDTA and/or taurolidine as detailed above, were tested by HPLC-MS.

b The indicated pyoverdines were investigated. Using the chromatograms, the different elution peaks were identified, and their areas were used for semiquantitative evaluation of each specific product. The fold change was calculated with respect to the corresponding SB control. The results shown are from a pool of three replicates of a representative experiment out of two performed.

c ND, not detectable.

Taken together, these HPLC-MS data indicated that EDTA and taurolidine treatment greatly impaired most of the Pseudomonas secretory potential. Such inhibitory effects were similar (no major differences) whether performing either 1 or 2 treatments; the impairment was transient or persistent, depending upon the secretory product assessed.

## DISCUSSION

Based on clinical experience with central venous catheter (CVC) infection treatment (38, 39) and according to a recently established in vitro model for biofilm assessment on medical devices (33), we report the anti-Pseudomonas efficacy of two lock solutions, EDTA and taurolidine, on PD catheters; both affect planktonic cells and PDC-associated biofilm, and even more, the secretory profile of the treated Pseudomonas biofilm is deeply hampered.

Pseudomonas often causes serious peritonitis in patients undergoing peritoneal dialysis (25). Challenges in treating these cases are based on the difficulty in eradicating infection. The pathogenic potential of this infectious agent is mostly mediated by its strong ability to produce biofilm and to release a plethora of secretory virulence products, in turn, tightly controlled by a sophisticated QS network (26–34). Here, using an engineered bioluminescent strain, we demonstrate that P. aeruginosa efficiently produces biofilm on silicone-based PD catheters *in vitro*; moreover, such biofilm can be affected to different extents by exposure to two traditional lock solutions, EDTA and taurolidine (alone or in combination), as assessed by real-time monitoring of both microbial growth and biofilm production on such medical devices.

EDTA and taurolidine are nonantibiotic solutions already used, in clinical practice, to prevent or treat central vein catheter-related bloodstream infections (38–40). Percival and Salisbury (24) have shown that tetrasodium EDTA is effective in reducing planktonic microbial load; moreover, it destroys the established biofilm with killing of embedded microorganisms. Taurolidine exerts its bactericidal activity by irreversible binding of its methylol groups to the bacterial cell wall and deeply affects microbial fimbriae and flagella, in turn reducing bacterial adhesion onto epithelial cells and abiotic surfaces (41–44). These antiadhesion properties of taurolidine combined with its direct killing activity likely lead to impairment of biofilm formation (44, 45). In line with the literature (46), we documented that taurolidine significantly reduces the viability of Pseudomonas planktonic cells. After just 1 h of treatment, the levels of bioluminescence signal sharply drop with all tested concentrations. Interestingly, a further reduction in microbial load occurs after 6 and 24 h of lock therapy. In contrast, EDTA shows a delayed inhibitory effect on Pseudomonas, detectable after 6 and 24 h of treatment. The combination of the two lock solutions returns data similar to those obtained using taurolidine alone, arguing against additive effects between the two products.

The two lock solutions also affect Pseudomonas biofilm that has been preformed *in vitro* on PDC pieces. As kinetically assessed, taurolidine causes a rapid abatement of bacterial load, which is evident within the first 2 to 3 h of treatment and with all tested doses. In contrast, EDTA significantly affects microbial load, but only at the highest concentration (2.5%). In parallel samples, the strict relation between BLI-based results and cell viability was confirmed by performing a CFU assay in selected samples (data not shown). Thus, in line with previous data from our group (33–35), the engineered Pseudomonas BLI strain provides a ductile and easy-to-manage model for *in vitro* real-time monitoring of microbial cell viability and growth on medical devices.

As shown by experiments where the contaminated PDC were treated, washed, and further incubated in fresh medium, the inhibitory effects of EDTA and/or taurolidine on Pseudomonas are transient. An intense microbial regrowth is observed in the 48- to 72-h time frame, independent of the treatment performed. It should be noted that the PDC-associated biofilms show different microbial loads at time 48 h because of the dissimilar antimicrobial activities exerted by the two lock solutions. Then, a similar and time-related bacterial regrowth occurs in all the samples; indeed, after 5 to 7 h, the RLU values reach plateau levels comparable among groups, including the control. Interestingly, despite such similar BLI values observed at 72 h, the amounts of biofilm produced at that time appear significantly different under the different conditions. The highest concentrations of EDTA (0.75% and 2.5%) or taurolidine (0.5%), or even the two together, significantly reduce the 72-h-old biofilm biomass. Furthermore, the increase in biofilm production detected at 0.125% taurolidine remains unexplained. Possibly, peculiar changes in cell density and/or bacterial metabolism, occurring under such conditions, may have facilitated biofilm formation. In any case, taken together, these findings suggest that the therapeutic effects of the lock solutions on the contaminated PDC mostly persist, at least in terms of antibiofilm activity.

To assess the effect of a repeated use of lock solutions, as happens in clinical practice (18) to prevent drug degradation during the management of CVC, the *in vitro*-contaminated PDC were exposed to a second treatment with EDTA/taurolidine solutions. As detailed above, the inhibitory effects show a trend similar to the one produced by the first treatment. In particular, microbial growth is deeply affected during treatment, and once again, a time-related recovery occurs upon drug removal. Likely, the presence of persister cells inside the treated biofilm may be responsible for prompting the regrowth observed after drug removal. Also, the impact of the 2nd lock solution treatment on the 96-h-old biofilm is significant, closely recalling the one observed after the 1st treatment. Unexpectedly, the removal of taurolidine at the highest dose, either alone or combined with EDTA, allows a further, yet transient, growth inhibition occurring between 102 and 114 h (taurolidine alone) or 102 and 106 h (taurolidine plus EDTA). The reasons for this fluctuating regrowth and its possible impact on further biofilm formation/persistence remain unexplored.

Taken together, these findings provide evidence that EDTA/taurolidine treatment transiently impairs microbial growth; also, biofilm production and persistence remain hampered to some extent. Thus, given the crucial role of biofilm in catheter-related infections, we envisage that the lock solutions may have a long-term action as anti-Pseudomonas tools through destabilization and/or destructuration of the sessile community.

The growing literature underlines the crucial involvement of secretory molecules, such as AI, in Pseudomonas virulence, given their role in QS regulation (26–32). Indeed, AI affect microbial adhesion and biofilm formation (30) and regulate the production of other key molecules (27), such as pyoverdines and phenazines. As siderophores, pyoverdines contribute to pathogenesis, mediating acquisition of key nutritional elements, while phenazines are involved in gene expression, bacterial fitness, and respiration (27, 30, 31). As a consequence, all these Pseudomonas secretory molecules have a key role in determining *in vivo* the course of the infection and the outcome of the disease (28, 29).

To add information on the events possibly involved in the EDTA and taurolidine-mediated biofilm impairment, we performed HPLC-MS analysis, providing the first evidence, to our knowledge, that the secretory profile of PDC-associated Pseudomonas biofilm is drastically affected by exposure to the two lock solutions. In particular, when comparing the peak areas of treated and untreated samples, the levels of the well-known AI, 3-oxo-C_12_-HSL, C_4_-HSL, PQS, and IQS, are deeply decreased when using EDTA and taurolidine, either alone or in combination. In particular, taurolidine appears slightly more effective than EDTA in reducing the levels of C_4_-HSL and PQS. Upon drug wash-out, the production of all such AI recovers up to 70 to 80% in relation to the untreated controls. Thus, in agreement with the microbial load/viability data, the Pseudomonas AI production is also impaired by these lock solutions and the effects once more are transient. In agreement with AI fluctuation, the levels of phenazine and pyocianin are deeply impaired after the first treatment and return to control levels upon drug removal in parallel with microbial regrowth; furthermore, they drop again after the second treatment. To a similar extent, the pyoverdine production drops to undetectable levels upon the first treatment; surprisingly, unlike all the other secretory products, 3 of these pyoverdines (Succ-p-Ser-Y, Succa-P-Ser-Y, and PyE) remain below the detection limit during the regrowth period, while PyD levels recover up to about 50% of those of the control. Upon the second treatment, all the tested pyoverdines consistently remain at undetectable levels. Based on these results, we speculate that EDTA and taurolidine solutions transiently affect AI and phenazine/pyocianin production by P. aeruginosa, while the production of pyoverdines appears irreversibly hampered. Whether this picture represents a fine regulation of different virulence factors remains to be established. In any case, the finding that EDTA and taurolidine may disturb Pseudomonas secretory potential, to a wide extent, calls for future *in vivo* studies on the clinical relevance of these lock solutions focusing on such novel parameters/targets.

Overall, our data provide *in vitro* evidence that taurolidine (at all the doses) and EDTA (at 2.5%), separately or in combination, deeply impact on two key virulence traits of Pseudomonas, namely, its propensity to produce biofilm on silicone catheters and its wide-spectrum secretory repertoire. In particular, taurolidine and EDTA, to a lesser extent, reduce microbial load and biofilm formation on PDC. Although not achieving eradication, these nonantibiotic solutions induce a biofilm biomass impairment, likely through destructuration of the matrix. Preliminary data on a Pseudomonas clinical isolate further strengthen the antibiofilm efficacy of the two lock solutions; indeed, as assessed by crystal violet assay, a 24-h exposure of the PDC-associated biofilm to EDTA, taurolidine, or both reduces microbial biomass by 51%, 66%, and 99%, respectively (data not shown). Whether these *in vitro* antimicrobial effects will have an *in vivo* counterpart, we may expect that treatment of the PDC with EDTA and taurolidine may result in reduced risk of catheter-related infections. Further studies will be needed to verify if such lock solutions might be combined with conventional antibiotics to increase the likelihood of clearing a PDC-associated infection as well as to verify peritoneal mesothelial cell tolerance to possible contact with the lock solution leaked from catheter.

### Conclusion.

Using an *in vitro* model, we provide new insights on the anti-Pseudomonas properties of taurolidine and EDTA. Though to different extents, they impair planktonic cell growth and biofilm production and persistence on catheters for peritoneal dialysis. Such inhibitory effects are transient; nevertheless, mass spectrometry analysis reveals that, upon treatment, the secretory armamentarium of P. aeruginosa biofilm is also deeply affected; in fact, the plethora of virulence factors commonly released by this pathogen are profoundly inhibited. These *in vitro* findings will open the way to further studies where lock solutions may be used to make the device-associated microbial biofilm more amenable to antibiotic treatment.

## MATERIALS AND METHODS

### Pseudomonas aeruginosa.

The bioluminescent P. aeruginosa strain P1242 (BLI-Pseudomonas) was used. As detailed elsewhere, BLI-Pseudomonas expresses the luciferase gene and luciferase substrate under the control of a constitutive P1 integron promoter (37); in particular, the viable cells constitutively emit measurable bioluminescence. The bioluminescent signal was measured at certain time points with a Fluoroskan reader (Thermo Fischer Scientific, Waltham, MA, USA); the values were expressed as RLU, a direct measure of the numbers of viable cells.

### Catheter-lock solutions.

Sodium calcium edetate (SALF; Pharmacological Laboratory, Bergamo, Italy) (EDTA) was used at the working concentrations of 2.5%, 0.75%, and 0.25%. Taurosept (Geistlich Pharma, Germany) (taurolidine), a catheter-lock solution containing 2% taurolidine as the antimicrobial component, was used at the working solutions of 0.5%, 0.25%, and 0.125% in saline buffer (SB). The working dilutions were prepared the day before each experiment. EDTA and taurolidine were tested alone or in combination against BLI-Pseudomonas.

### P. aeruginosa culture and growth conditions.

Bacteria rescued from −80°C glycerol stocks were initially seeded onto tryptic soy agar (TSA) plates and incubated overnight at 37°C; then, a fresh single colony was collected, inoculated into 10 ml of tryptic soy broth plus 2% sucrose (TSB) and cultured overnight at 37°C. The culture was then washed (centrifuged twice) and inoculated into fresh medium (TSB). In order to evaluate the bacterial concentration, 100 μl of the microbial suspension was seeded in a 96-well plate, and the optical density was measured at 595 nm (OD_595_) using the spectrophotometer Tecan Sunrise. Through a reference curve, the OD values were converted to CFU/ml. For all the experiments, the starting bacterial suspension was adjusted at 10^5^ CFU/ml in TSB.

### Peritoneal dialysis catheter (PDC) preparation.

First, 24 h before each experiment, a sterile disposable peritoneal dialysis catheter (PDC) (Argyle Covidien Manfield, MA, USA) was cut under sterile conditions into pieces of 0.5-cm length, as previously described (33). A maximum of 6 PDC pieces per tube were placed into 1.5-ml microcentrifuge tubes, covered with fetal bovine serum (FBS), and incubated at 37°C overnight in static conditions prior to being used in the experiments detailed below.

### Taurolidine MIC assay.

The MIC assay was performed using the broth microdilution method according to the CLSI M7-A6 Standards (36). Accordingly, the commercially available taurolidine solution was serially diluted and tested at the final dosages in the wells, ranging from 0.5% to 0.0075%. In parallel, gentamicin (2 mg/ml) was included as the positive control. A bacterial cell suspension (5 × 10^5^ cells/ml in TSB plus 2% sucrose, obtained from overnight cultures) was seeded (100 μl/well) in a 96-well U-bottom microtiter plate; then, the bacterial cells were exposed to saline buffer or treated with scalar doses of taurolidine solution (100 μl/well). Then, the plate was incubated at 37°C for 24 h. The MIC was defined as the lowest concentration that inhibited visible Pseudomonas growth.

### Protocols for assessing EDTA and taurolidine effects on BLI-Pseudomonas growth, adhesion, and biofilm formation on PDC pieces.

Different protocols were used to assess EDTA and/or taurolidine effects on several parameters concerning BLI-Pseudomonas, at various time points, as detailed in the paragraphs below.

**Protocol for assessing planktonic BLI-Pseudomonas** **cells.** BLI-Pseudomonas (10^5^/ml, 100 μl/well) was seeded in 96-well plates in SB (control) or in the presence of EDTA and/or taurolidine at the concentrations indicated elsewhere. The plate was incubated at 37°C for 1 h, 6 h, or 24 h, and the bioluminescent signal was measured at each time with a Fluoroskan reader (Thermo Fischer Scientific, Waltham, MA, USA); the RLU values represented the amounts of live cells in the treated and untreated groups.

**FIG 4:**
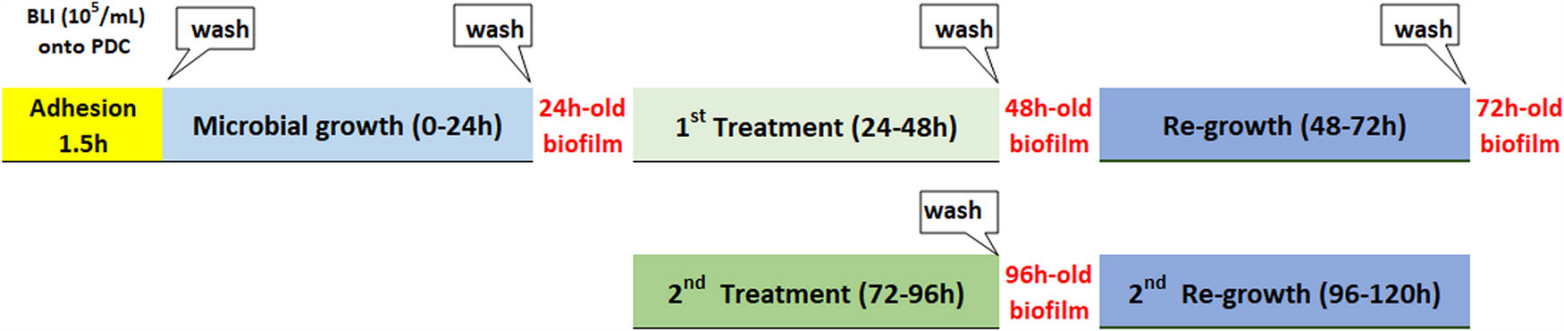
Flow chart of the protocol for assessing taurolidine and/or EDTA effects on PDC-associated biofilm. PDC pieces (0.5 cm; pretreated overnight with FBS) were exposed to BLI-Pseudomonas (10^5^/ml) for 1.5 h, washed and incubated in fresh medium (0 to 24 h) to allow biofilm formation. After PDC washing, the 1st treatment with the lock solutions was performed (24 to 48 h). During that time, microbial growth was kinetically measured and biofilm was assessed after PDC washing (48 h). The PDC were further incubated in fresh medium to allow microbial regrowth (48 to 72 h). Then, the 2nd treatment with the lock solutions was performed (72 to 96 h). During that time, microbial growth was kinetically measured, and biofilm was assessed after PDC washing (96 h). Finally, the PDC were further incubated in fresh medium to allow microbial regrowth (96 to 120 h). Additional details are given in Materials and Methods.

**Protocol for measuring microbial adhesion, growth, and biofilm formation.** We investigated the effects of EDTA and/or taurolidine on Pseudomonas growth in the presence of PDC and its ability produce biofilm on such abiotic surfaces. The biofilm-harboring PDC were treated once or twice with EDTA and/or taurolidine and kinetic analyses were performed, as summarized in the flow-chart of the study (Fig. 4).

### Microbial adhesion (1.5 h), growth and biofilm formation (24 h) on PDC pieces.

BLI-Pseudomonas (10^5^/ml in TSB, 180 μl/well) was seeded in 96-well plates containing 1 PDC piece/well. The plates were incubated at 37°C for 90 min (adhesion time). Then, the PDC pieces were washed once with SB at room temperature (RT) and transferred into new wells and the RLU were measured to establish the adhesion. Afterwards, the PDC pieces (immersed in TSB, 180 μl/well) were incubated at 37°C and the BLI signal was detected to establish microbial growth, hourly up to 24 h. Later, the PDC pieces were washed with SB at RT and transferred to new wells (containing 180 μl of TSB) and the BLI signal was measured to quantify the 24-h-old biofilm.

**Exposure of the 24-h-old biofilm to EDTA and/or Taurolidine (24-48h).** The PDC, harboring a 24-h-old biofilm, were treated with 100 μl of SB, EDTA, and/or taurolidine (at different concentrations) and incubated at 37°C for additional 1 h, 6 h, or 24 h. The RLU were measured hourly, and at the end of the 24-h treatment, the PDC pieces were washed and transferred to new wells, and the BLI signal was assessed again as a measure of the 48-h-old biofilm.

**Regrowth (48 to 72 h) of EDTA and/or taurolidine-treated biofilm.** The PDC, harboring 48-h-old treated and untreated biofilms, were immersed in fresh medium (180 μl/well) and incubated for an additional 24 h at 37°C, the BLI signal was again measured every hour. At the end, the PDC pieces were washed and transferred to new wells and the bioluminescence was measured to establish the amounts of 72-h-old biofilm.

**Exposure of the 72-h-old biofilm to a 2nd treatment with EDTA and/or taurolidine.** The PDC pieces, harboring 72-h-old (treated and untreated) biofilms, were exposed to a retreatment (2nd treatment) with SB, EDTA and/or taurolidine another 24 h at 37°C; during that time, the microbial growth was kinetically measured up to 96 h by BLI assay. Then, the PDC pieces were washed and transferred to new wells, and the biofilm (96-h-old) was measured again. Subsequently, fresh medium (180 μl/well) was added and PDC pieces were incubated for another 24 h at 37°C to kinetically evaluate the microbial regrowth up to 120 h.

### High-performance liquid chromatography-mass spectrometry (HPLC-MS).

HPLC-MS analysis was performed at the University CIGS center, as extensively detailed elsewhere (34, 35). In particular, selected supernatants from samples where Pseudomonas biofilm-harboring PDC had been treated or not treated with EDTA and taurolidine (1st and 2nd treatment) were analyzed for the presence of the following molecules: 3-oxo-C_12_-HSL, C_4_-HSL, PQS, IQS, pyoverdines, and pyocyanins. Prior to being spectrometrically tested, the supernatants were filtered on Amicon Ultra-0.5 10 K centrifugal filter devices and diluted 1:5 with 5% methanol and 0.2% formic acid in Milli-Q water. The instrument used for the HPLC-MS analysis was an UltiMate 3000 system, consisting of an online degasser, a binary pump (HPG 3400RS), a well plate autosampler (WPS; 3000RS), and a thermostatted column compartment (TCC; 3000RS) coupled to a Q Exactive hybrid quadrupole-orbitrap mass analyzer via a HESI-II heated electrospray ion source (Thermo Scientific). Chromatographic separation of a 5-μl sample injection was performed on a Poroshell 120 SB-C18 100 × 2.1-mm ID, 2.7-μm particle size (ps) column (Agilent) at 30°C and a 0.4 ml/min flow rate. A linear gradient elution scheme was used with mobile phase components of 0.1% formic acid in water (A) and methanol (B). The gradient started at 2% B, which was maintained for 0.5 min and then was increased to 30% B in 30 min and up again to 98% B in 24.5 min. The column was then kept at 98% B for 17.9 min, and then the starting conditions were restored in 0.1 min and maintained for 19 min pending successive injections. Electrospray ionization was operated in positive ion mode, using nitrogen as sheath gas (50 arbitrary units), auxiliary gas (290°C, 40 arbitrary units), and sweep gas (3 arbitrary units). The sprayer voltage was kept at 3.8 kV, and the transfer capillary temperature was set at 320°C. The Q Exactive device was operated in full mass spectrometry (MS) data-dependent MS2 (MS/ddMS2) mode. The full MS scan range was set at *m/z* 170 to 1,000 at 70,000 full width half maximum (FWHM) resolution (*m/z* 200). The automatic gain control (AGC) target was set at 1.0 × 10^6^ with a maximum injection time (IT) of 200 ms. dd-MS2 acquisitions at 17,500 FWHM resolution (*m/z* 200) were triggered for the top 3 precursor ions following each full MS scan. The intensity threshold for precursor ion selection was set to 1.0 × 10^5^, and then dynamic exclusion was active for 20.0 s. The AGC target and maximum IT for the MS2 experiments were set to 2.0 × 10^5^ and 50 ms, respectively. Each precursor ion was fragmented using stepped normalized collision energy (NCE) values at 28, 50, and 75.

Compound elution provided peaks appearing in their specific chromatographic traces, showing the abundance of their respective charged ions over the chromatographic run (mass range chromatograms). Using the Thermo Fisher FreeStyle program, the peak areas (adimentional values provided by the software) were used for semiquantitative evaluation, as detailed elsewhere (34).

### Statistical analysis.

Quantitative variables were tested for normal distribution. Statistical differences between treated and untreated samples were analyzed according to Student’s *t* test using GraphPad Prism 8. The values in Fig. 1 to 3 and Table 1 are the mean ± standard error of the mean (SEM) of 6 to 8 replicate values obtained from two independent experiments. The data shown in Tables 2, 3, and 4 are from a representative experiment, where a pool of triplicates for each condition was processed and analyzed using HPLC-MS.
